# High LIFr expression stimulates melanoma cell migration and is associated with unfavorable prognosis in melanoma

**DOI:** 10.18632/oncotarget.4688

**Published:** 2015-07-25

**Authors:** Hongwei Guo, Yabin Cheng, Magdalena Martinka, Kevin McElwee

**Affiliations:** ^1^ Department of Dermatology and Skin Science, University of British Columbia, Vancouver, Canada; ^2^ Department of Dermatology, Affiliated Hospital of Guangdong Medical College, Zhanjiang, Guangdong, China; ^3^ Department of Pathology, University of British Columbia, Vancouver, British Columbia, Canada

**Keywords:** LIFr, melanoma, biomarker, cell migration

## Abstract

Increased or decreased expression of LIF receptor (LIFr) has been reported in several human cancers, including skin cancer, but its role in melanoma is unknown. In this study, we investigated the expression pattern of LIFr in melanoma and assessed its prognostic value. Using tissue microarrays consisting of 441 melanomas and 96 nevi, we found that no normal nevi showed high LIFr expression. LIFr staining was significantly increased in primary melanoma compared to dysplastic nevi (*P* = 0.0003) and further increased in metastatic melanoma (*P* = 0.0000). Kaplan–Meier survival curve and univariate Cox regression analyses showed that increased expression of LIFr was correlated with poorer 5-year patient survival (overall survival, *P* = 0.0000; disease-specific survival, *P* = 0.0000). Multivariate Cox regression analyses indicated that increased LIFr expression was an independent prognostic marker for primary melanoma (*P* = 0.036). LIFr knockdown inhibited melanoma cell migration in wound healing assays and reduced stress fiber formation. LIFr knockdown correlated with STAT3 suppression, but not YAP, suggesting that LIFr activation might stimulate melanoma cell migration through the STAT3 pathway. Our data indicate that strong LIFr expression identifies potentially highly malignant melanocytic lesions at an early stage and LIFr may be a potential target for the development of early intervention therapeutics.

## INTRODUCTION

When diagnosed early, melanoma is treatable with surgical excision and patients can remain relapse free for up to 10 years [1]. An in-depth understanding of the biology underlying melanoma initiation and progression could allow for improved staging and subtype classification, and might lead to the development of better therapeutic agents and interventions [2]. However, molecular markers that enable high risk patients to be identified during the early stages of melanoma progression still remain elusive [3].

Leukemia inhibitory factor (LIF), an IL-6 family member, is a pleiotropic cytokine which plays roles in cell proliferation and differentiation [4, 5]. For example, LIF is used to maintain murine embryonic stem (ES) cell pluripotency through promotion of self-renewal and suppression of stem cell differentiation [6]. LIF also has a potentially significant role in adult skin homeostasis and in hyperproliferative skin disorders [7].

Cancer is increasingly viewed as a stem cell disorder where signaling pathways that normally promote self-renewal of stem cells drive carcinogenesis [8–10]. LIF signaling is expressed at elevated levels in a broad range of human cancers, including melanoma [4, 5, 11–13]. Notably, overexpression of LIF is significantly associated with a worse relapse-free survival rate in breast cancer patients [14] and the amount of LIF secreted appears to regulate tumorigenesis [12, 13]. Constantly enhanced expression of LIF in skin cancers, including melanoma, have been reported [15, 16]. In adenomas, LIF promotes proopiomelanocortin (POMC) synthesis [17], which mediates HPA (hypothalamic-pituitary-adrenal) axis response to stress [18, 19]. Melanomas produce higher levels of LIF [16] and POMC [20], suggesting LIF may stimulate melanoma growth in part by promoting HPA axis peptides [21, 22].

Since LIF signals through formation of heterodimers between a specific LIF receptor (LIFr) and the common IL-6 family co-receptor gp130 [23], a strong rationale exists to investigate the expression and potential role of LIFr in melanoma tumorigenesis. LIFr was recently identified as a significant prognostic factor in human breast carcinoma [24], and blockade of LIFr inhibits the chemotaxis of rhabdomyosarcoma cells [13], suggesting that LIFr activation may promote metastasis and increase the invasive potential of solid tumors.

Here, we found that LIFr expression was significantly increased in different stages of human melanocytic lesions and LIFr was an independent prognostic factor for survival of melanoma patients. Studies *in vitro* suggested that knockdown of LIFr expression inhibited melanoma cell migration through STAT3 (signal transducer and activator of transcription 3) suppression rather than YAP (Yes-associated protein) signaling pathways.

## RESULTS

### Clinicopathologic features of the tissue microarrays

Tissues from a total of 713 patients were incorporated into tissue microarrays (TMAs). However, 441 melanoma (292 primary melanomas and 149 metastatic melanomas) and 96 nevi (35 normal nevi and 61 dysplastic nevi) were evaluated for LIFr staining in this study because of biopsy core loss or insufficient cells present in the TMA core sections. For the 441 melanoma cases, 259 were male and 182 were female, with ages ranging from 7 to 95 years (median, 60 years). Melanoma staging was completed in accordance with the American Joint Committee on Cancer (AJCC) stages. In all, 182 tumors were at AJCC stage I, 110 were at stage II, 61 were at stage III, and 83 were at stage IV, 5 cases were at an uncertain stage. Among the 292 primary melanoma cases, 137 were thinner than 2.0 mm, 101 were thicker than 2.0 mm and 54 were *in situ*; 85 cases were located in sun-exposed areas (head and neck), whereas 205 were located in sun-protected sites, and 2 were from an unspecified location (supplementary Table S1).

### Enhanced LIFr expression is positively correlated with melanoma progression

**Figure 1:**
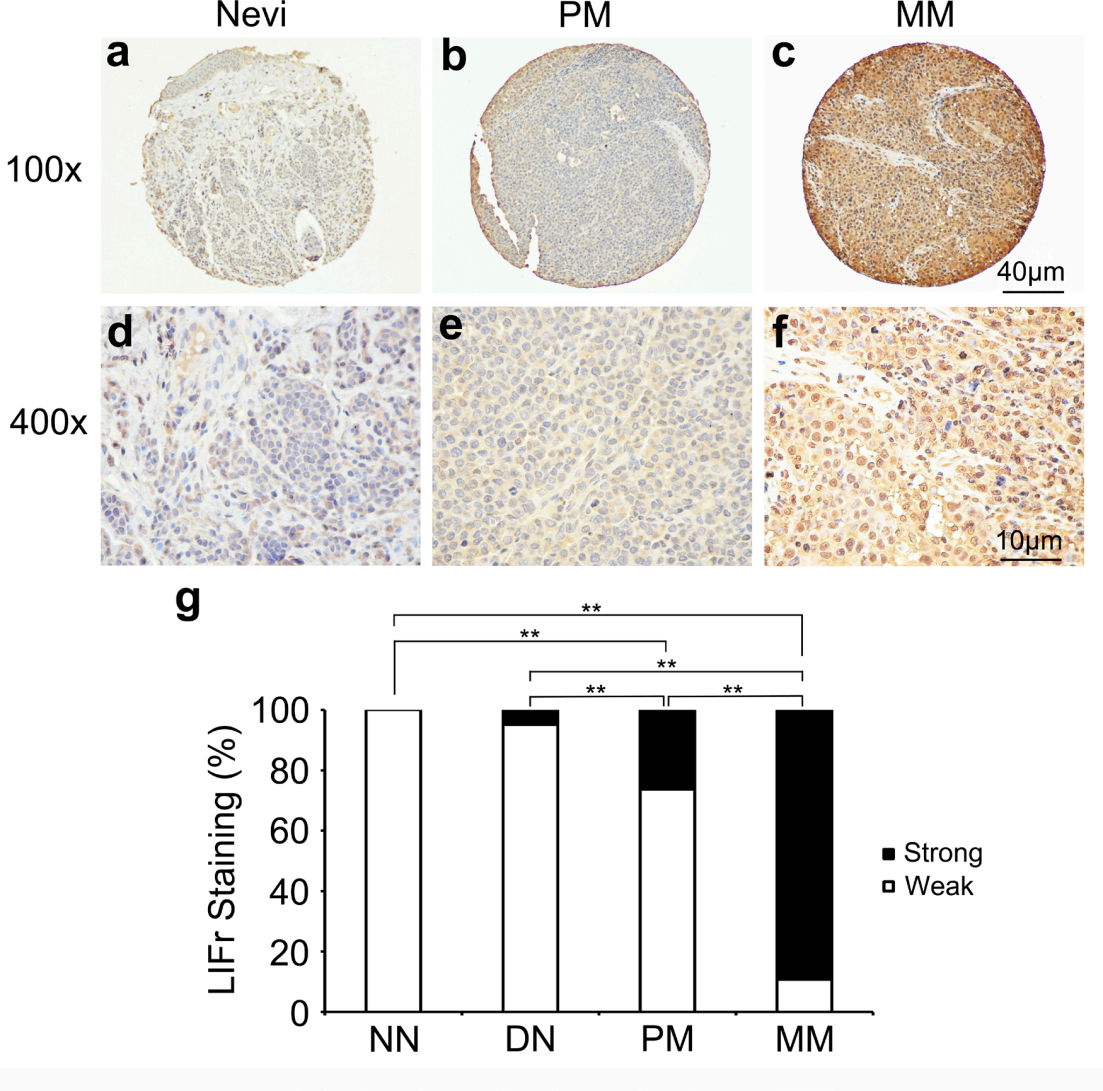
LIFr expression is increased in human advanced melanoma Representative images of LIFr immunohistochemical staining in nevi, primary melanoma and metastatic melanoma TMA. **a, d.** Normal vevi with negative staining. **b, e.** primary melanoma with moderate positive staining. **c, f.** Metastatic melanoma with strong positive staining. LIFr expression was significantly increased from nevi to melanoma. (a-c) Bar = 40 μm; (d-f) bar = 10 μm. **g.** Increased LIFr expression correlates with melanoma progression. LIFr expression was enhanced in primary melanoma compared with dysplastic nevi (*P* = 0.0003, χ2 test) and further increased in metastatic melanoma compared with primary melanoma (*P* = 0.0000, χ2 test). **P < 0.01. DN, dysplastic nevi; LIFr, leukemia inhibitory factor receptor; MM, metastatic melanoma; NN, normal nevi; PM, primary melanoma.

Immunohistochemical LIFr labeling of normal nevi, dysplastic nevi, primary melanomas, and metastatic melanomas was performed on TMA slides (Figure 1 and supplementary Figure S1). The LIFr staining was predominantly in the cytoplasm and therefore only cytoplasmic staining was evaluated. The specificity of the LIFr antibody was examined by immunofluorescence and Western blot analysis (Figure 4). The anti-LIFr antibody utilized recognizes epitopes at the C-terminus of LIFr [25]. The functionally important LIFr C-terminus contains five tyrosine residues and several Y*X X*Q motifs that are genetically highly conserved [26]. A specific LIFr blocking peptide completely abolished anti-LIFr antibody immunoreactivity confirming the specificity of the staining reaction (Supplementary Figure S2). Among the groups, no normal nevi showed strong LIFr expression, LIFr staining was significantly more common in primary melanoma, with a subset exhibiting strong staining, compared to dysplastic nevi (*P* = 0.0003, χ^2^ test), and expression was further increased in metastatic melanoma (*P* = 0.0000, χ^2^ test) (Figure 1). These results suggested that increased LIFr expression is correlated with malignant melanocytic lesion progression and melanoma metastasis.

### Increased LIFr expression is correlated with patient age, tumor thickness and ulceration in primary melanomas; and gender and AJCC stages in all melanomas

**Figure 2:**
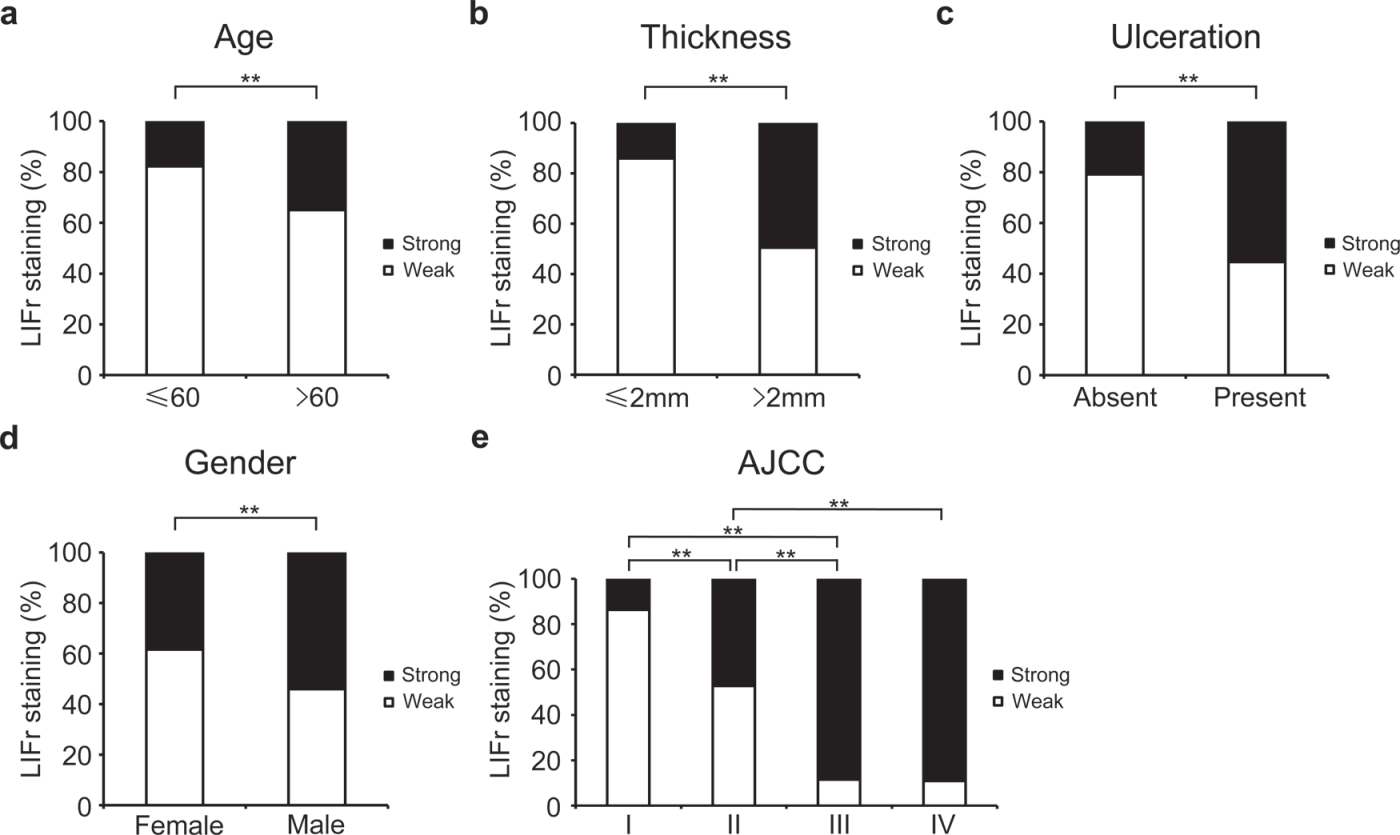
LIFr expression is associated with patient age, tumor thickness and ulceration in primary melanoma patients and gender and AJCC in all melanoma patients **a.** The high LIFr expression was significantly more frequent in primary melanoma patients aged over 60 years old (*P* = 0.009; χ2 test); **b.** patients with tumor thickness greater than 2.0 mm (*P* = 1e-8; χ2 test); **c.** melanoma with ulceration (*P* = 8.8e-7; χ2 test). **d.** In all melanoma patients, the high LIFr expression was significantly more frequent in males than females (*P* = 0.001; χ2 test); **e.** In all melanoma the percentage of cases with high LIFr expression was significantly increased in AJCC stages III and IV (*P* = 0.0000, χ2 test). *P < 0.05, **P < 0.01.

We examined the correlation between cytoplasmic LIFr staining and the patients' clinicopathologic characteristics. High LIFr expression was significantly more frequent in patients aged over 60 years (34.9%) as compared to patients aged less than 60 years (16.8%) (*P* = 0.0090, χ^2^ test); high LIFr expression was significantly more common in patients with tumor thickness greater than 2.0 mm (49.5%) compared with tumors thinner than 2.0 mm (15.3%) (*P* = 1e-8; χ^2^ test). The percentage of cases with high LIFr expression was also increased in melanoma tissues with ulceration (55.3%) compared to melanoma tissues without ulceration (20.8%) (*P* = 8.8e-7; χ^2^ test). Moreover, the percentage of cases with high LIFr expression was significantly enhanced in male patients (*P* = 0.0012, χ^2^ test) and patients in AJCC stages III and IV (88.9%) compared to females and patients in stages I and II (26.4%) (*P* = 0.0000, χ^2^ test) (Figure 2). Nodular melanoma, which has metastatic potential [27], exhibited significantly higher LIFr expression compared with superficial spreading melanoma and lentigo malignant melanoma (*P* = 0.0000 and 0.0002 respectively) (supplementary Table S1). We did not observe any significant correlation between cytoplasmic LIFr staining and tumor location (supplementary Table S1).

### Increased LIFr expression is associated with poor survival of melanoma patients

**Figure 3:**
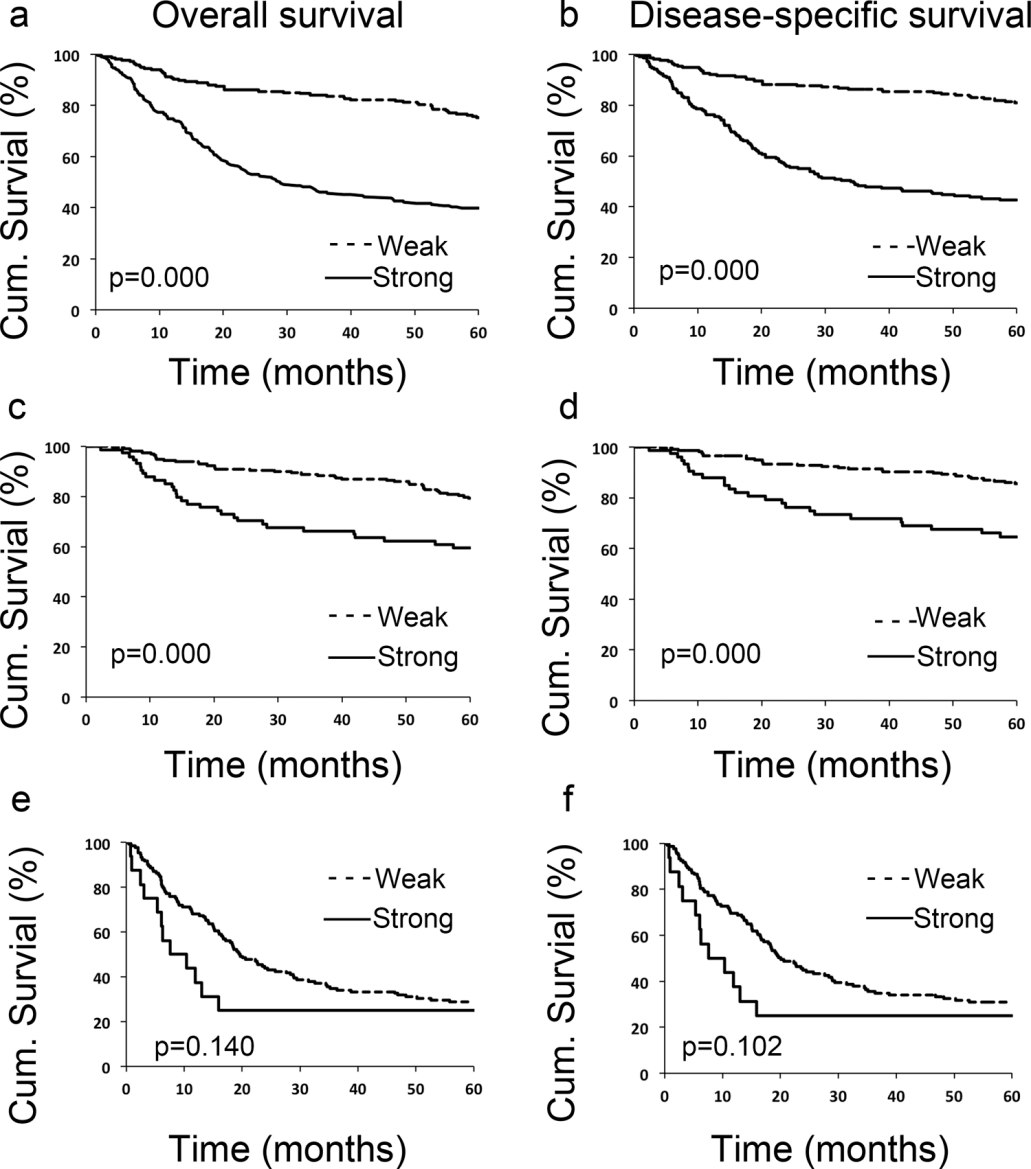
LIFr expression is significantly correlated with 5-year survival of all melanoma patients and primary melanoma patients Patients with strong LIFr expression have a significantly worse overall **a, c.** and disease specific 5-year survival **b, d.** than those with weak staining in all melanoma patients (including primary and metastatic melanoma) and primary melanoma patients (*P* = 0.000 and 0.000 respectively, log-rank test). However LIFr expression did not show any correlation to the survival rate of metastatic melanoma patients **e, f.** (*P* = 0.140 and 0.102 respectively, log-rank test).

To evaluate the potential correlation between LIFr cytoplasmic expression and 5-year patient survival, we constructed Kaplan–Meier survival curves using overall, or disease-specific 5-year survival data. In all melanoma samples the mean overall 5-year survival in the low LIFr expression group was 75.6% compared to 39.6% in the high LIFr expression group; a significant difference by log-rank analysis (overall survival, *P* = 0.0000; disease-specific survival, *P* = 0.0000, log-rank test) (Figure 3). Multivariate Cox proportional hazards regression analysis showed that LIFr expression predicted both overall and disease-specific patient survival (*P* = 0.0384 and 0.0312, respectively) (Table 1). To investigate if LIFr expression was correlated with patient survival at specific melanoma stages, the patients were divided into primary and metastatic melanomas and the patient survival was analyzed. Primary melanoma patients with strong LIFr expression had worse mean overall, and disease-specific, patient survival compared to patients with low LIFr expression (overall survival, *P* = 0.0000; disease-specific survival, *P* = 0.0000, log-rank test) (Figure 3). Multivariate analysis revealed LIFr expression has less prognostic power than ulceration and thickness, but higher power than age and gender (overall survival, *P* = 0.0878; disease-specific survival, *P* = 0.036) (Table 1). High or low LIFr expression did not show correlation to the survival rate of metastatic melanoma patients (Figure 3), therefore the multivariate analysis was not performed in these cases.

**Table 1:** Multivariate Cox proportional regression analysis on 5-year overall and disease specific survival of 292 primary and 149 metastatic melanoma patients

	Overall survival					Disease-specific survival				
Variables	^+^β	SE	HR	95% CI	*P*	^+^β	SE	HR	95% CI	*P*

All Melanoma (*n* = 441)										
age	−0.297	0.154	0.743	0.55–1.00	0.054	−0.195	0.165	0.823	0.60–1.14	0.237
sex	−0.167	0.160	0.846	0.62–1.16	0.296	−0.226	0.170	0.798	0.57–1.11	0.185
AJCC	−1.238	0.206	0.290	0.19–0.43	<0.001	−1.435	0.227	0.238	0.15–0.37	<0.001
LIFr	−0.456	0.220	0.634	0.41–0.98	0.038	−0.532	0.247	0.588	0.36–0.95	0.031

Primary Melanoma (*n* = 292)										
age	−0.734	0.267	0.480	0.28–0.81	0.006	−0.530	0.302	0.589	0. 33–1.06	0.079
sex	−0.275	0.247	0.760	0.47–1.23	0.266	−0.325	.0286	0.722	0.41–1.27	0.255
thickness	−1.150	0.297	0.317	0.18–0.57	0.000	−1.399	0.365	0.247	0.12–0.51	0.000
ulceration	−0.483	0.287	0.617	0.35–1.08	0.092	−0.678	0.321	0.508	0.27–0.95	0.035
LIFr	−0.436	0.256	0.646	0.65–1.07	0.088	−0.614	0.293	0.541	0.31–0.96	0.036

Coding of variables: Age was coded as 1 (≤60 years) and 2 (>60 years). Sex was coded as 1 (male) and 2 (female). Thickness was coded as 1 (thinner than 2.0 mm) and 2 (thicker than 2.0 mm). Ulceration was coded as 1 (with ulceration) and 2 (without ulceration). LIFr was coded as 1 (low expression) and 2 (high expression).

+ β regression coefficient.

Abbreviations: SE, standard error of β; HR, hazard ratio; CI, confidence interval.

### The expression of LIFr is increased in melanoma cell lines

**Figure 4:**
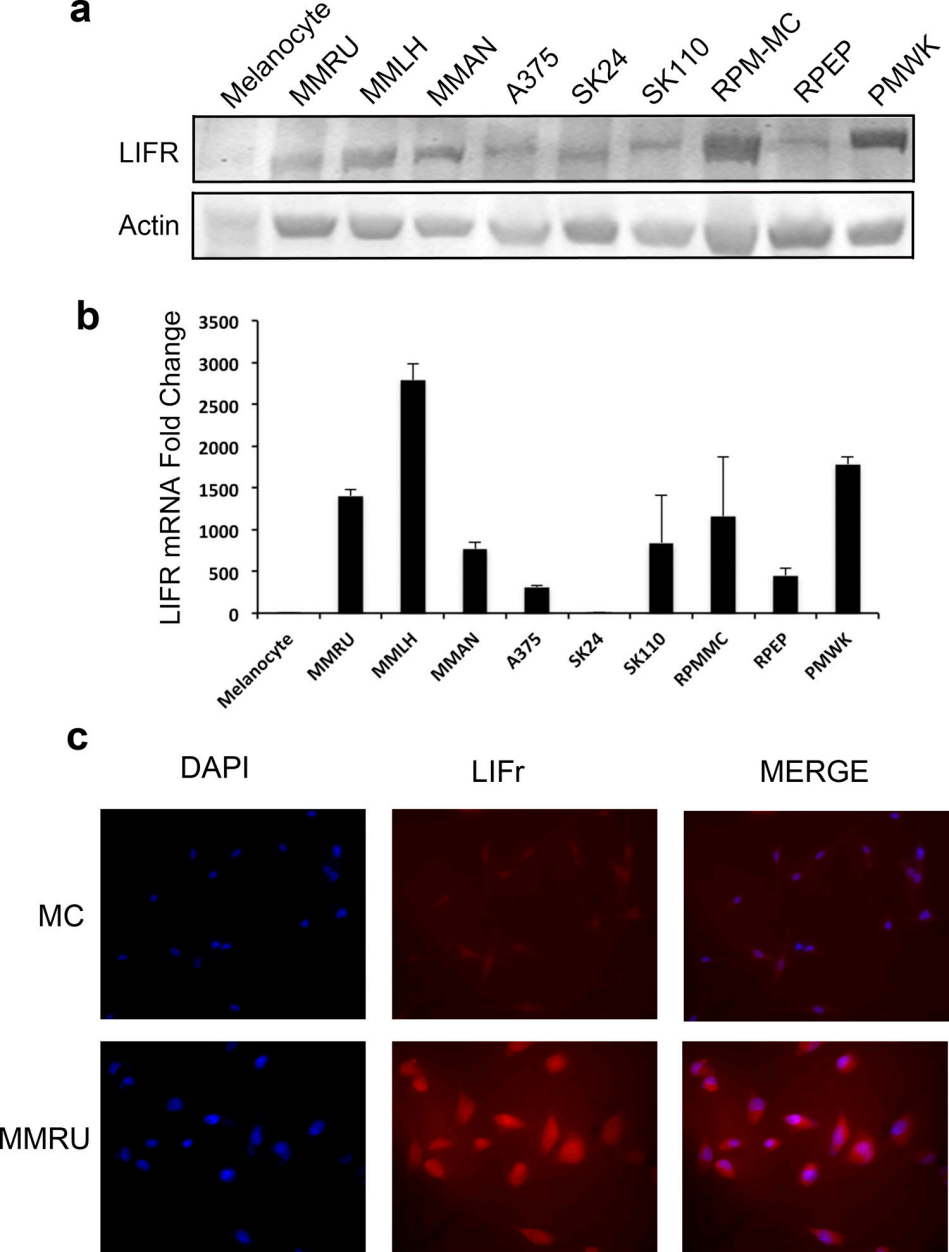
LIFr protein and mRNA expression are enhanced in melanoma cell lines compared with melanocytes Whole cell extracts were obtained from normal human melanocytes and melanoma cell lines for Western blot **a.** and real-time reverse transcription quantitative PCR analysis **b.** Fold change in melanoma cell lines is relative to melanocytes as the baseline comparator (set to value of 1 fold). Bars equal to means ± SD. **c.** Immunofluorescence LIFr staining of melanoma cell line MMRU and melanocytes (MC).

We examined LIFr expression in different melanoma cell lines and normal melanocytes by Western blot and reverse transcriptase–quantitative PCR (qPCR). Our data showed that most melanoma cell lines expressed higher LIFr protein and mRNA compared to normal melanocytes. Also, the immunofluorescence staining for representative melanoma cell line MMRU was much stronger than the normal melanocyte cell line (Figure 4). The increased LIFr protein and mRNA expression in melanoma cell lines was consistent with TMA observations.

### LIFr does not influence the proliferation of melanoma cells

Since LIFr expression was increased in melanoma when compared to nevi, and LIF signaling is linked to proliferation of breast [11], kidney, prostate [4] and pancreas carcinoma cells [5], we hypothesized that LIFr expression affects melanoma cell growth. The MMRU and PMWK cells, which express high LIFr, were transfected with LIFr siRNA to knock down LIFr expression, and the cell growth was analyzed. Over a 48 h time course, cells treated with LIFr siRNA had no significant reduction in growth rates compared to negative siRNA (Neg siRNA) treated control cells (Supplementary Figure S3). The results indicate that LIFr does not significantly affect the growth rate of melanoma cells.

### LIFr promotes melanoma cell migration

**Figure 5:**
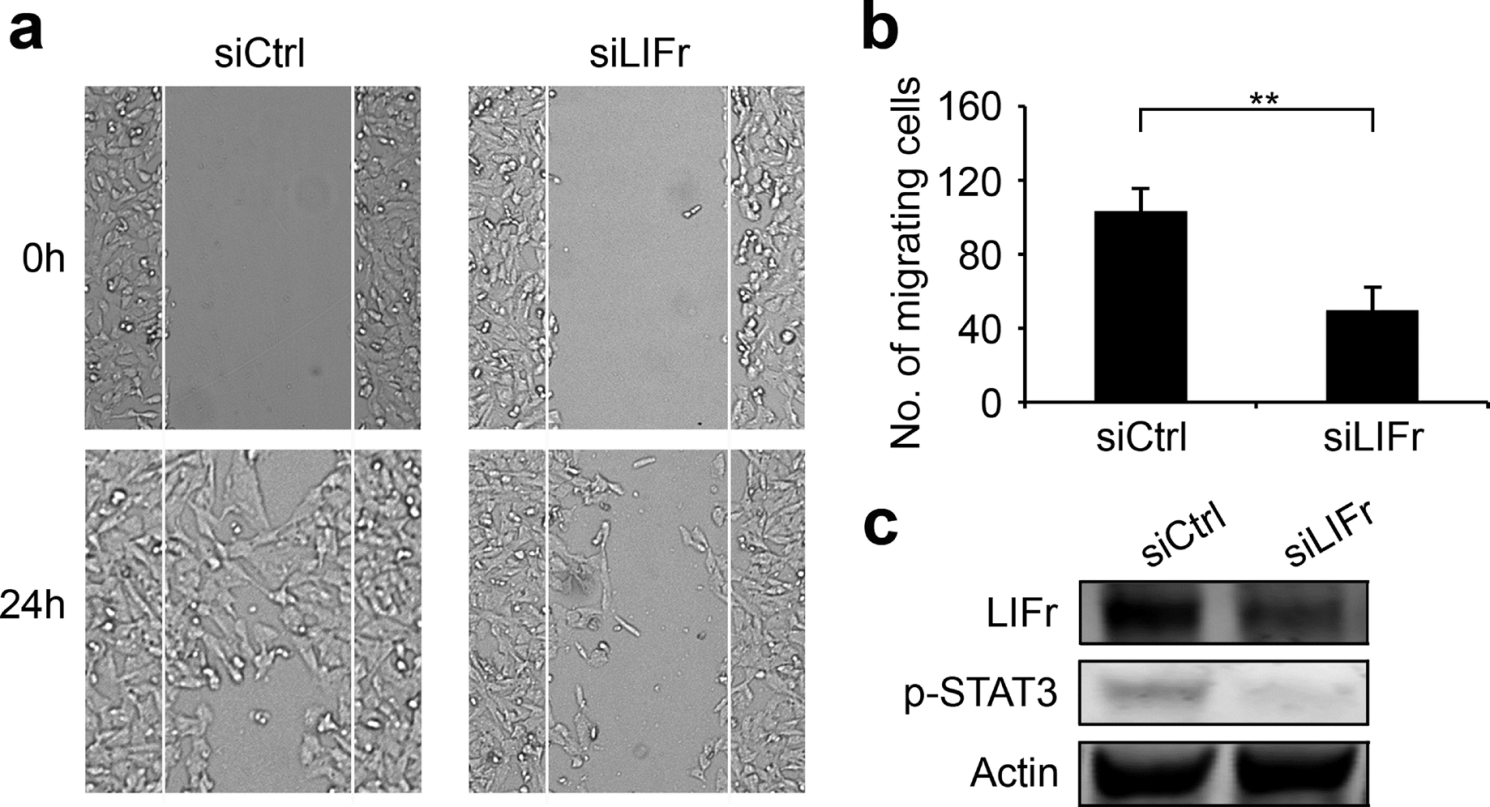
Knockdown of LIFr inhibits melanoma cell migration **a.** Representative images of the effects of LIFr knockdown on melanoma cell migration. **b.** The effects of LIFr expression inhibition on melanoma cell migration was quantified by counting the migrated cells in five random fields of each well; Bars equal to means ± SD. The data were obtained from three independent experiments. **P < 0.01. **c.** Western blot analysis of LIFr expression; knockdown of LIFr inhibited phosphorylated STAT3 (p-STAT3) expression.

As LIFr expression progressively increases from primary melanoma to metastatic melanoma, and LIFr activation regulates metastatic behavior [13], we investigated whether LIFr affects melanoma cell migration and invasion. In wound healing assays, LIFr knockdown caused 52% reduction in MMRU melanoma cell migration as compared with the control group (Figure 5). Boyden chamber invasion assays showed LIFr knockdown tended to inhibit MMRU cell invasion, but did not reach statistical significance (Supplementary Figure S4).

### LIFr knockdown in melanoma cells reduces STAT3 signaling rather than Hippo-YAP signaling

**Figure 6:**
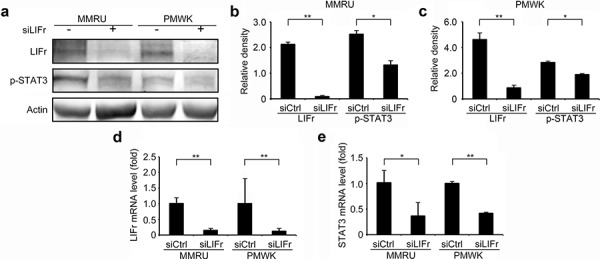
Knockdown of LIFr decreases STAT3 mRNA levels and phosphorylated STAT3 expression Forty-eight hours after transfection, cells were plated for migration assays, and the remaining cells were harvested for LIFr and STAT3 mRNA expression detection and Western blot analysis. **a.** Representative images of LIFr, phosphorylated STAT3 (p-STAT3) and b-actin expression in Western blotting. Analyses of band density are presented as the relative ratio of LIFr and STAT3 to actin for MMRU cells **b.** and PMWK cells **c, d.** LIFr mRNA expression in MMRU and PMWK cell lines after LIFr knockdown relative to controls (set to value of 1 fold). **e.** STAT3 mRNA expression in MMRU and PMWK cell lines after LIFr knockdown relative to controls (set to value of 1 fold). The data was analyzed by Student's *t*-test, Bars equal to mean ± SD. All experiments were carried out in triplicate. *P < 0.05, **P < 0.01.

Activated LIFr stimulates the JAK/STAT (Janus kinase/signal transducer and activator of transcription) and MAPK cascades [28]. It has been shown that LIFr inhibits breast cancer metastasis by inactivating the activity of the transcriptional co-activator YAP [24, 29, 30]. We hypothesized that the three pathways might be the signaling mechanism(s) by which LIFr impacts melanoma cell migration. We did not detect significant changes in YAP expression after LIFr knockdown (supplementary Figure S5). However, STAT3 mRNA was significantly reduced and the expression of phosphorylated STAT3 at tyrosine 705 was decreased with LIFr knockdown (Figure 6). p38 mRNA was diminished but phosphorylated p38 reduction has not reached the statistical significance (supplementary Figure S5). Therefore knockdown of LIFr may reduce melanoma cell migration via inhibition of STAT3, partly involving p38.

### LIFr knockdown in melanoma cell lines reduces MMP2 function

**Figure 7:**
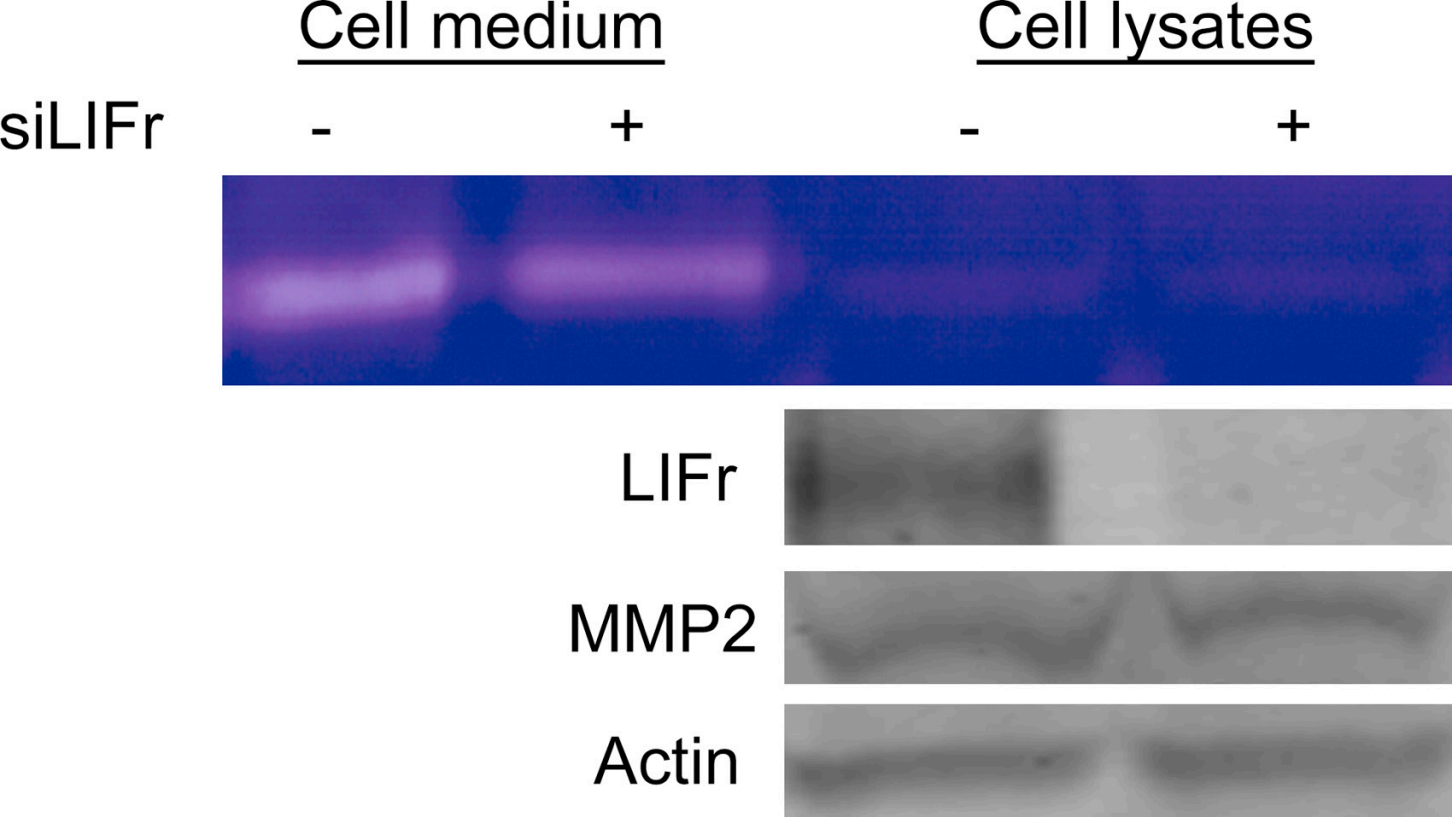
Knockdown of LIFr reduces MMP2 activation Forty-eight hours after transfection with siRNAs, then serum-free medium starved 24 hours, the proteins in the conditioned medium and MMRU cell lysates were concentrated and extracted; zymography assay and Western blot analysis were performed. The active MMP2 extracted from MMRU cell lysates showed little change, but the active MMP2 from MMRU cell conditioned medium was reduced. Western-blot showed that there were no significant changes in Pro-MMP2 protein expression.

Cancer cell migration is regulated by integrins, matrix-degrading enzymes, cell–cell adhesion molecules and cell–cell communication. Matrix metalloproteinase 2 (MMP2) aids tumor cell migration by digesting the extracellular matrix surrounding the tumor cells [31]. Strong MMP2 expression is associated with worse melanoma patient survival and is an independent prognostic factor for primary melanoma [32]. Since both MMP2 and LIFr could influence primary melanoma patient survival, and up-regulation of MMP2 plays a crucial role in melanoma cell migration [33], we investigated the correlation between MMP2 and LIFr expression. Although Western-blot showed no significant changes in Pro-MMP2 protein expression after LIFr knockdown, zymography assays showed that MMP2 function was reduced (Figure 7). LIFr knockdown reduces melanoma cell migration in part by reduced activation of MMP2.

### LIFr knockdown reduces stress fiber formation during melanoma cell migration

**Figure 8:**
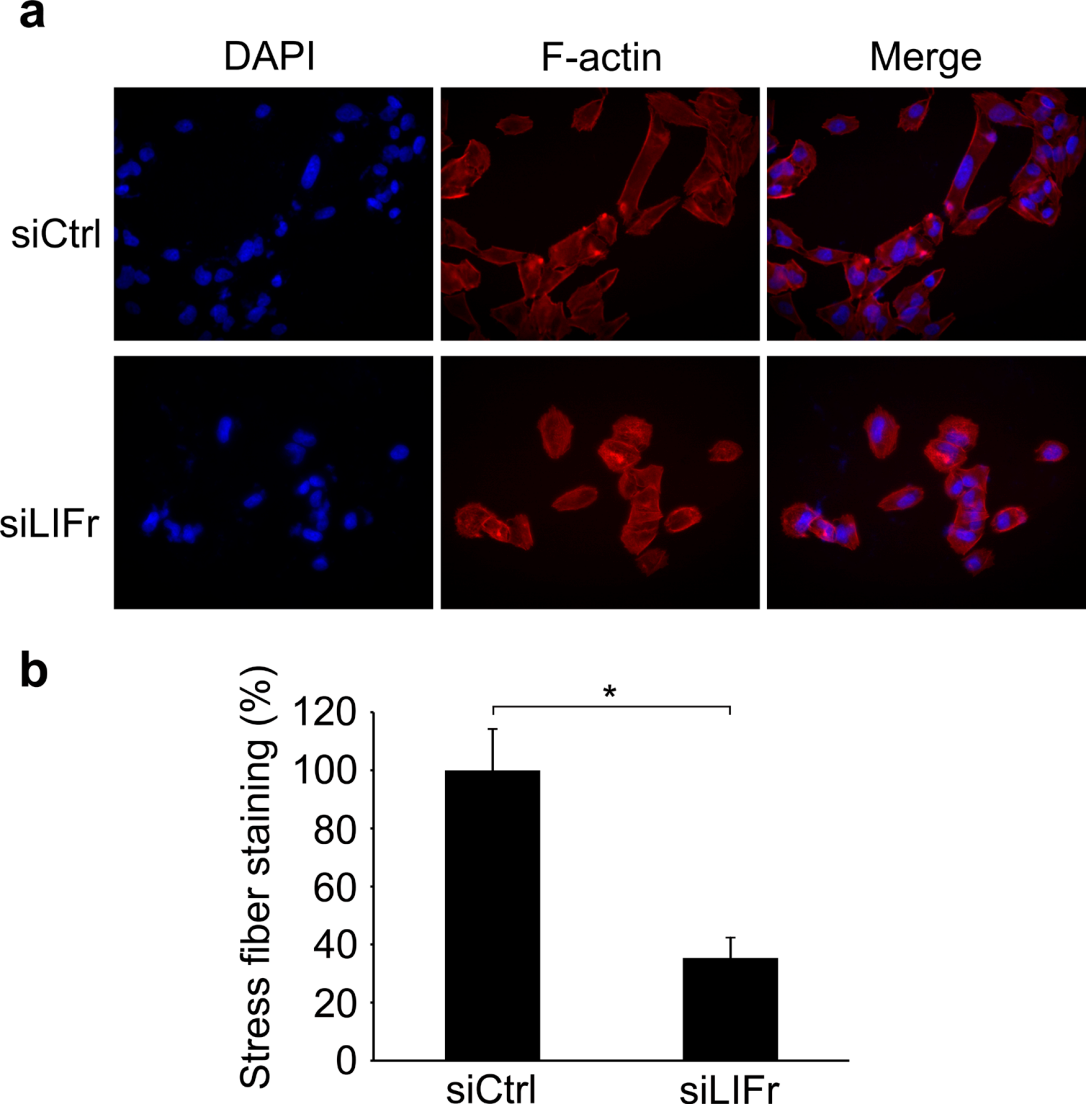
Knockdown of LIFr inhibits actin stress fiber induction **a.** Representative images of stress fiber formation in LIFr knockdown and control melanoma cells. Forty-eight hours after transfection with siRNAs, cells were seeded on cover slips at a density of 2 × 10^4^ cells per well in six-well plates for 24 h. Cells seeded on coverslips were serum starved overnight followed by serum stimulation with 10% fetal bovine serum for 1 h, then the cells were stained with rhodamine-conjugated phalloidin. Serum-induced stress fibers were thinner, poorly oriented and less spread in LIFr knockdown MMRU cells, whereas there were thicker, well-aligned stress fibers running across the control MMRU cells. Data were obtained from triplicate experiments. **b.** Quantification of stress fiber staining intensity. There was a significant reduction of stress fiber formation in LIFr knockdown MMRU cells compared with control MMRU cells.

The actin cytoskeleton has a fundamental role in cell migration and abnormalities in actin dynamics are associated with cancer cell transformation [34]. The disruption of F-actin cyto-architecture is required for melanoma cell migration [35–37]. We observed that LIFr knockdown significantly reduced stress fiber formation after serum stimulation (Figure 8b), there was a lower degree of cell spreading in LIFr knockdown cells compared with the well-aligned stress fibers in cells transfected with control siRNA (Figure 8a).

## DISCUSSION

Our data using TMA technology showed that there was no strong LIFr expression in normal nevi; LIFr was significantly increased in primary melanoma, and further increased in metastatic melanoma, compared to dysplastic nevi. There are a paucity of molecular markers that can accurately distinguish between nevus and melanoma [38]. From our results, we propose that increased LIFr expression in nevi may serve as an early alarm signal for the transformation from nevus to melanoma. We did not observe significant correlation between LIFr expression and tumors in sun exposed and sun protected areas. Our finding implies that LIFr may not be directly involved in UV mediated melanocyte transformation in which the disturbance of melanin activities and melanogenesis play a distinct role [39, 40].

The AJCC staging system describes the extent of disease progression in melanoma patients [41]. Gender factors other than stage at diagnosis and body site reduce mortality risk for female melanoma [42, 43]. Increasing tumor thickness is highly correlated with decline in 5- and 10-year patient survival rates; survival rates of patients with an ulcerated melanoma are proportionately lower than those of patients with a non-ulcerated melanoma of equivalent T category [44]. The age of patients with primary melanoma correlates significantly with survival, though the degree of correlation is much lower compared with those for melanoma thickness and ulceration [45, 46]. Nodular melanoma has greater metastatic potential [27] compared with superficial spreading melanoma and lentigo malignant melanoma. We found that increased LIFr expression correlated with patients aged over 60 years, thick tumors, tumor ulceration, and nodular melanoma in primary melanoma. Higher LIFr expression was also associated with melanoma in males, and AJCC stages III and IV in all patients with melanoma. Moreover, multivariate Cox regression analysis revealed that LIFr expression is an independent factor for predicting disease-specific 5-year survival in primary melanoma, but not metastatic melanoma patients. This indicates that LIFr may be important in early melanoma progression; high LIFr expression could identify subgroups of high-risk melanoma patients with reduced chances of survival at an early stage. Our proposal is consistent with a previous study which suggested that LIFr signaling is important in the early stages of prostate cancer progression [47].

LIFr is the specific receptor of LIF, but Oncostatin M (OSM), cardiotrophin-1 (CT1) and ciliary neurotrophic factor (CNTF) can also bind LIFr. Membrane-bound LIFr (gp190) prefers to form a heterodimer by combining with a common IL-6 receptor subunit (gp130) rather than assemble a homodimer by combining with another LIFr [48, 49]. The formation of this complex results in the activation of the receptor-associated Janus kinases (JAKs) to recruit STAT3. When bound to the receptor, STAT3 molecules are phosphorylated on tyrosine 705 (Tyr705) residues and dimerize with another phosphorylated STAT3. The dimers are then translocated to the nucleus where they bind to promoters and enhancer regions of their target genes [50]. Dimerized LIFr not only phosphorylates STAT3 to signal JAK/STAT, but also phosphorylates SHP2 to activate SHP2/Ras/MAPK cascades [51, 52]. Also, protein kinase C (PKC) and PI3-kinase pathways are activated via LIFr [53]. The involvement of these signaling pathways is well known in melanoma progression [54]. LIFr signaling is upstream of the Hippo-YAP pathway in breast cancer metastasis [24, 29, 30]. We sought to investigate the influence of LIFr on these signaling cascades in melanoma cells.

Although LIFr can diminish YAP phosphorylation and induce its cytoplasmic retention in breast cancer cells [24], suppressing LIFr in melanoma cells did not inhibit YAP phosphorylation or decrease YAP mRNA expression. Instead, eliminating LIFr in melanoma cells markedly reduced STAT3 mRNA expression and inactivated STAT3 by inhibiting its phosphorylation. STAT3 phosphorylation promotes oncogenesis in a variety of tissues including melanoma [55, 56] and represents a valid target for novel drugs [57]. STAT3 mRNA levels are increased and STAT3 phosphorylation is enhanced in adenocarcinomas [58]. LIFr co-receptor gp130 is essential for STAT3 phosphorylation and activation of the STAT3 gene [59]. Since LIFr forms heterodimers with gp130, potentially knockdown of LIFr may reduce gp130 function and decrease STAT3 mRNA levels and STAT3 phosphorylation.

Further, LIFr is required for STAT3 activation to induce myeloid leukemia cell differentiation and growth arrest; the cytoplasmic domain of LIFr is capable of STAT3 signal transduction even when LIFr forms a homodimer [28, 60]. The most distal motifs of the LIFr C-terminus can induce myeloid differentiation of leukemia cells by enhancing STAT3 phosphorylation [26]. Therefore, LIFr can signal via the STAT3 pathway as long as the LIFr C terminus is present. Potentially, splicing events may occur to form isoform variants [61] which may explain the dual LIFr specific bands we sometimes observed by Western blot and as seen in other cancers [24]. Our data suggests that LIFr enhances STAT3 production and activation consequently modifying melanoma cell features. However, the specific constitution of the receptor complex and the exact cellular nature of STAT3/LIFr expression in melanoma cells needs further investigation.

STAT3 signaling is required for cell motility and represents an essential effector in regulating actin cytoskeleton reorganization [55, 62]. Knockdown of STAT3 selectively inhibits IL-6 stimulated cell migration by localization in focal adhesion complexes [63]. Consequently, we hypothesized that LIFr affects stress fiber formation and subsequently influences melanoma cell migration. We found that silencing LIFr significantly suppressed melanoma cell migration, and LIFr knockdown reduced stress fiber formation and melanoma cell motility.

Matrix-degrading enzyme MMP2 can regulate tumor cell migration by digesting extracellular matrix surrounding tumor tissues [31]. In general, MMP2 is secreted in latent form and is activated on the cell surface [33]; integrins localize active MMP2 on the surface of invasive melanoma cells [64]. LIF modifies melanoma cell capacity to adhere to matrix components by upregulation of integrin expression [65]. LIFr may or may not have effects on MMP2 mediated tumor progression depending on the cell types evaluated [66–68]. After LIFr knockdown, the active MMP2 consolidated from melanoma cell conditioned medium was reduced, indicating that LIFr helps activate MMP2. The mechanism of LIFr signaling in cell migration is far more complicated beyond regulation of MMP2 activation and actin stress fiber induction and warrants further investigation.

In summary, we found that increased LIFr expression is significantly correlated with melanoma progression and LIFr is an independent factor for predicting disease-specific 5-year survival in primary melanoma patients. Loss of LIFr expression significantly inhibited melanoma cell migration and inhibited STAT3 phosphorylation. Based on these findings, LIFr may be used as a prognostic marker of patient survival and blocking LIFr activity may be a potential therapeutic approach for malignant melanoma.

## MATERIALS AND METHODS

### Study approval

The use of human skin tissues and the waiver of specific patient consent in this study were approved by the Clinical Research Ethics Board of the University of British Columbia (CREB study ID H09–01321). The study was conducted according to the principles expressed in the Declaration of Helsinki.

### Immunohistochemistry of tissue microarrays

The construction of a melanoma tissue microarray (TMA) was described previously [69]. The immunohistochemical labeling of TMA slides was performed as described [32]. Anti-human-LIFr rabbit polyclonal antibody (C19, 1:100 dilution, Santa Cruz Biotechnology, Dallas, TX, USA), was used. The LIFr antibody was tested for specificity using a specific LIFr blocking peptide (LIFr C-19 P; Santa Cruz) and protein blot analysis (Supplemental Figure S2). Negative controls for array labeling were performed by omitting the LIFr antibody.

### Evaluation of immunostaining

The evaluation of LIFr staining was done blinded and independently by one dermatopathologist and two other observers. The percentage of LIFr positive cells was scored as 1 (1–25%), 2 (26–50%), 3 (51–75%) and 4 (76–100%). Label intensity was also scored on a 4-point system, a score of 0 was given for complete absence of staining, 1 for weak staining, 2 for medium staining, and 3 for strong staining. The product of intensity and percentage positive cells was taken as the immunoreactive score (IRS), based on the IRS score system as published [70]; LIFr staining was defined as low expression (IRS 0–6) or high expression (IRS 8–12).

### Immunofluorescence analysis

Immunofluorescent staining was described previously [71]. Melanocytes (MC) and MMRU cells were grown on cover slips in a six-well plate for 24 h then fixed with 2% paraformaldehyde. The cover slips were incubated with primary antibody (anti-human-LIFr rabbit polyclonal antibody, 1:100 dilution, Santa Cruz) overnight at 4°C and Goat anti-Rabbit Alexa Fluor^®^ 568 conjugated secondary antibody (1:750 dilution, Life Technologies Inc, Burlington, ON, USA) for 1 h. For f-actin staining, after serum stimulation, we stained the cells with rhodamine-conjugated phalloidin. Stained cells and stress fibers were quantified by Image J software (NIH) from photos taken randomly in 10 fields. Five cells were quantified in each field. A region was drawn around each cell to be measured, and the same size region was drawn in an area without fluorescent objects to be used for background subtraction. Corrected total cell fluorescence (CTCF) = Integrated Density- (Area of selected cell x Mean fluorescence of background readings).

### Cell culture and transfection

Human metastatic and primary melanoma cell lines, MMRU and PMWK, were cultured in DMEM supplemented with 10% fetal bovine serum in 5% CO_2_ at 37°C. Cells were grown to 70% confluency before siRNA transfection. LIFr expression silencing was achieved with two LIFr siRNA oligonucleotides (human LIFR siRNA, sc-35808, Santa Cruz; siLIFR ID: SASI_Hs02_00330115, Sigma-Aldrich, St Louis, MO, USA) at a final concentration of 20 nM and 150 nM respectively, using Silenfect transfection reagent (Bio-Rad, Mississauga, ON, Canada) as per manufacturer's instructions. Forty-eight hours after transfection, cells were plated for proliferation, migration and invasion assays, and the remaining cells were harvested for mRNA expression detection and Western blot analysis.

### Western blot analysis

Whole-cell lysates were prepared from the cell lines for Western blotting as previously described [69]. The following antibodies were used: anti-human LIFr rabbit polyclonal antibody (Santa Cruz) and, for confirmation, anti-human LIFr alpha biotinylated affinity purified polyclonal antibody (R&D Systems, Minneapolis, MN, USA), anti-human phospho-STAT3 (Tyr705) rabbit monoclonal antibody, anti-human phospho-YAP rabbit monoclonal antibody, or anti-human phospho-MAPK p38 rabbit polyclonal antibody (all Cell Signaling Co, Beverly, MA, USA), anti-human MMP2 mouse monoclonal antibody (Biolegend, San Diego, CA, USA) and anti-human b-actin (Sigma). The protein visualization and imagine analysis was performed on an Odyssey infrared imaging system (LI-COR Biosciences, Lincoln, NE, USA).

### Real-time reverse transcription quantitative PCR

Total RNA was prepared by Qiazol extraction (Qiagen, Venlo, Limburg, Netherlands) and reverse transcribed into cDNA with the Transcriptorc DNA Synthesis System (Applied Biological Materials, Richmond, BC, Canada). Real-time qPCR was performed with SYBR Green Master mix system (Roche, Mississauga, ON, Canada). Primers for LIFR, STAT3, YAP and MAPK are shown in supplementary Table S2.

### Gelatinolytic zymography assay

Gelatinolytic zymography was performed as previously described [35]. Briefly, 48 h after transfection with siRNAs, serum-free medium was used to starve the cells for 24 h. The proteins in the conditioned medium were concentrated with YM-30 centricon membranes (Millipore, Billerica, MA, USA) at 3, 500 rpm for 4 h at 4°C. Proteins (10 μg) were loaded in non-reducing conditions on a 10% polyacrylamide gel containing 0.1% gelatin (Sigma). After electrophoresis was performed, SDS was removed from the gel by incubation in Triton X-100 exchange buffer [20 mmol/L Tris-HCl (pH 8.0), 150 mmol/L NaCl, 5 mmol/L CaCl, and 2.5% Triton X-100] for 60 min followed by a 3 × 10 min wash with the incubation buffer (same buffer without Triton X-100). Gelatinolytic activities were developed in incubation buffer 40 h at 37°C, stained with 0.5% Coomassie blue R250 (Sigma) for 1 h and destained with 30% methanol and 10% glacial acetic acid for 1 h.

### Monolayer wound healing assay

A wound healing assay was conducted as described [36]. Forty-eight hours after transfection with siRNAs, a standard 200 μl pipette tip was drawn across the well to produce a wound. The monolayers were washed to remove floating cells and incubated in fresh complete medium for another 24 h. Photographs were taken at the same position of the wounds at 0 and 24 h time points. The starting wound edges were defined in each photo by black lines based on the scratch at the 0 h time point and the numbers of cells migrating across these lines were counted [36]. The experiments were performed three times in triplicates.

### Cell invasion assay

Cell invasion analysis was done using Boyden chamber assays [69]. 20 μl of 5 mg/ml Matrigel (BD Biosciences) in serum-free medium was added to the upper compartment of 24-well Transwell culture chambers (with 8.0 μm pore size polycarbonate membranes). After transfection 48 h, MMRU and PMWK cells (4 × 10^4^) suspended in 250 μl of serum-free medium were seeded in the upper compartment and 750 μl of complete medium was added to the lower compartment. After 24 h incubation, cells were fixed with 10% trichloroacetic acid at 4°C for 1 h. Any non-invaded cells were removed from the upper surface of the filter carefully with a cotton swab. Invaded cells on the lower side of the filter were stained with 0.5% crystal violet for 2 h at room temperature or mounted in Permount mounting media. The invaded cells on the filter were counted under a light microscope or a fluorescence microscope and/or the retained dye on the filters was extracted by 30% acetic acid, followed by reading the absorbance at 590 nm. The experiments were performed in triplicates.

### Sulforhodamine B (SRB) cell growth assay

To compare cell growth rates, cells were seeded in 24-well plates 48 h after transfection with siRNA. At each time point, cells were fixed with 10% trichloroacetic acid, stained with 0.4% sulforhodamine B in 1% acetic acid, and then destained with 1% acetic acid. Cell density was quantified by dissolving bound dye in 10 mmol/L Tris (pH 10.5) followed by colorimetric determination at 550 nm. The initial time point (baseline) was measured by fixing cells immediately after they had attached to the tissue culture plate, 6 h after seeding. Subsequent time points were measured by fixing cells 24 and 48 h later. Relative rates of cell growth were calculated as a ratio of the cell density at each time point over the cell density at baseline. The data are the averages of results from three separate experiments performed with triplicates.

### Statistical analysis

Correlations between LIFr and clinicopathologic parameters were evaluated by Kruskal Wallis test and χ^2^ test between patient subgroups. Survival time was calculated from the date of melanoma diagnosis to the date of death or last follow-up. The Kaplan-Meier method and log-rank test were performed to evaluate the effect of LIFr expression on the overall and disease-specific survival. The Cox proportional hazards regression models was used for multivariate analysis. *P*-value <0.05 was considered as statistically significant. All the statistical analysis was performed using SPSS version 11.5 (SPSS, Chicago, IL, USA) software.

## SUPPLEMENTARY FIGURES AND TABLES



## ABBREVIATIONS

AJCC: American Joint Committee on Cancer
CT1: Cardiotrophin-1
CNTF: Ciliary neurotrophic factor
IRS: Immunoreactive score
JAK/STAT: Janus kinase/signal transducer and activator of transcription
HPA: Hypothalamic-pituitary-adrenal
LIF: Leukemia Inhibitory Factor
LIFr: Leukemia Inhibitory Factor receptor
MMP2: Matrix metalloproteinase 2
MAPK: Mitogen- activated protein kinase
Neg siRNA: Negative siRNA
OSM: Oncostatin M
POMC: Proopiomelanocortin
PKC: Protein kinase C
SRB: Sulforhodamine B
TMA: Tissue microarray
Tyr705: Tyrosine residue 705
YAP: Yes-associated protein
