# Supplementary material for: High LIFr expression stimulates melanoma cell migration and is associated with unfavorable prognosis in melanoma
Source: Oncotarget. 2015 Jul 25;6(28):25484–98. doi: 10.18632/oncotarget.4688 (PMC4694846; doi:10.18632/oncotarget.4688)
Supplement: Supplementary file 1 [file oncotarget-06-25484-s001.pdf]

SUPPLEMENTARY FIGURES AND TABLES

Supplementary Table S1. LIFr staining and clinicopathologic characteristics of 441 melanoma patients

Variables	LIFr staining			
	**Low**	**High**	**Total**	***P***
All melanoma (*n* = 441)				
**Age** (years)				
<=60	103(49.3)	106(50.7)	209	0.1844
>60	129(55.6)	103(44.4)	232	
**Sex**				
Male	119(45.9)	140(54.1)	259	0.0012
Female	112(61.5)	70(38.5)	182	
**AJCC**				
I	157(86.3)	25(14.7)	182	0.0000
II	58(52.7)	52(47.3)	110	
III	7(11.5)	54(88.5)	61	
IV	9(10.8)	74(89.2)	83	
Primary melanoma (*n* = 292)				
**Age**				
<=60	120(82.2)	26(16.8)	146	0.0009
>60	95(65.1)	51(34.9)	146	
**Sex**				
Male	111(71.2)	45(28.8)	156	0.3037
Female	104(76.5)	32(23.5)	136	
**Tumor thickness**				
In situ	48(88.9)	6(11.1)	54	1e-8
<=2.0 mm thick	116(84.7)	21(15.3)	137	
>2.0 mm thick	51(50.5)	50(49.5)	101	
**Ulceration**				
Absent	194(79.2)	51(20.8)	245	8.8e-7
Present	21(44.7)	26(55.3)	47	
**Subtype**				
Acrolentigous	4(57.1)	3(42.9)	7	0.0001
Lentigous	57(81.4)	13(18.6)	70	
Nodular	22(48.9)	23(51.1)	45	
Superficially spreading	90(82.6)	19(17.4)	109	

**Variables**		**LIFr staining**		
	**Low**	**High**	**Total**	***P***
Spindle cell type	4(50.0)	4(50.0)	8	
Unspecified	36(67.9)	17(32.1)	53	
**Lymphocytic response**				
Absent	135(70.7)	56(29.3)	191	0.1157
Present	80(78.4)	21(21.6)	102	
**Site**				
Sun-exposed	60(70.6)	25(29.4)	85	0.4241
Sun-protected	154(75.1)	51(24.9)	205	
Unspecified	1(50.0)	1(50.0)	2	
Metastatic melanoma (*n* = 149)				
**Age**				
<=60	8(9.3)	78(80.7)	86	0.5081
> 60	8(12.7)	55(87.3)	63	
**Sex**				
Male	8(4.4)	95(95.6)	103	0.0796
Female	8(17.4)	38(82.6)	46	

Supplementary Table S2. Sequences of the Primers Used for qPCR

	Forward	Reverse
LIFR	5′-CACCTGGTCTTGCGAGCCTA-3′	5′-AGCCACTGCCACTGGGATGA-3′
STAT3	5′-GCCAGAGAGCCAGGAGCA-3′	5′-TGAAGCTGACCCAGGTAGCGCTGC-3′
YAP	5′-GTTTGGATGATGGATGCCATT-3′	5′-ATGCTGTGACATGAAGCATCTGA-3′
MAPK(P38)	5′-GTGCCCGAGCGTTACCAGAAC-3′	5′-CTGTAAGCTTCTGACATTTC-3′
b-actin	5′-CCCTGAGGCACTCTTC-3′	5′-AGGTCTTTGCGGATGT-3′

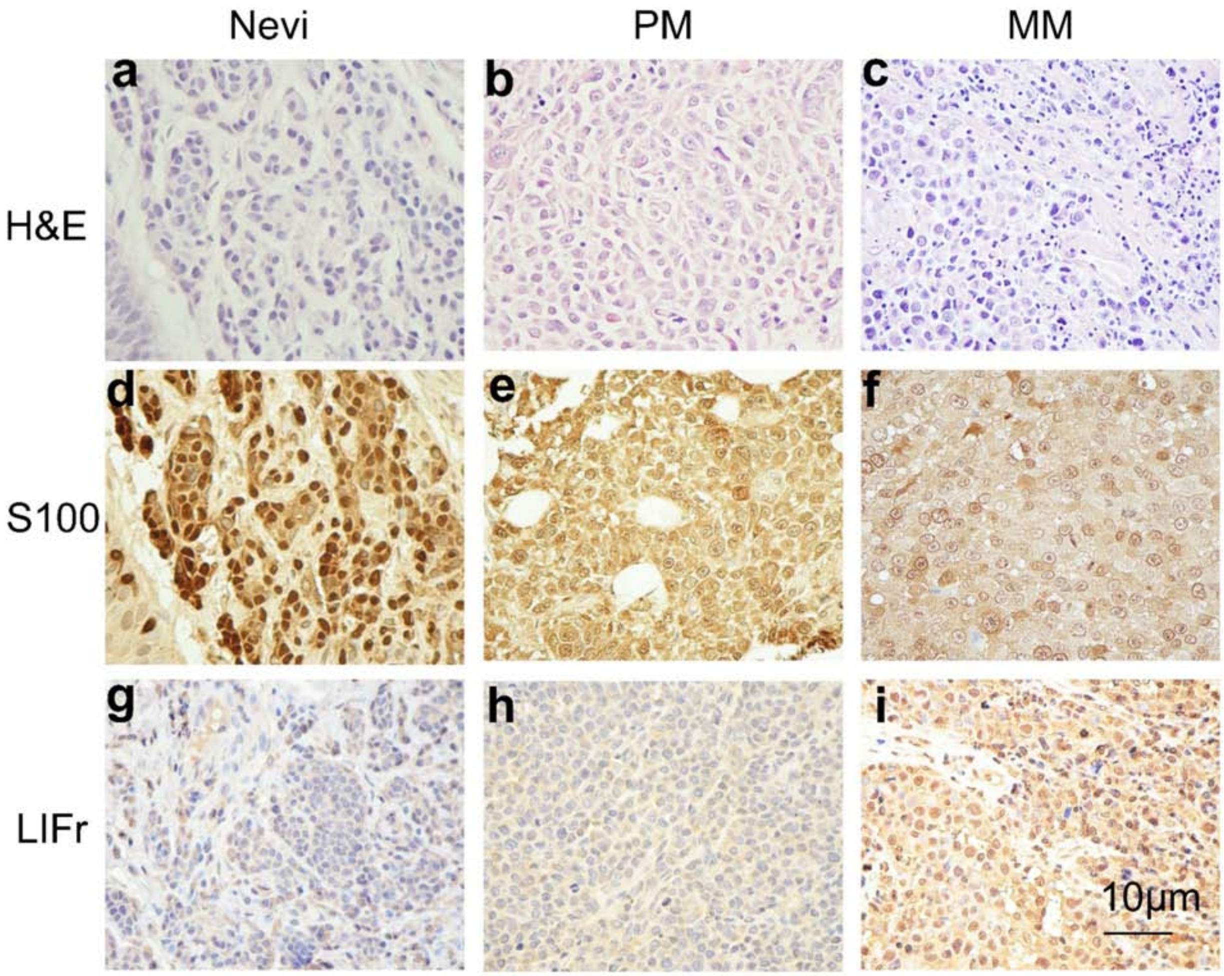

Supplementary Figure S1: Histological feature of different melanocytic lesions. a–c. H&E staining in nevi, primary melanoma and metastatic melanoma; **d–f.** S100 labeling in nevi, primary melanoma and metastatic melanoma; **g–i.** Representative images of LIFr immunohistochemical staining in nevi (negative staining), primary melanoma (weak staining) and metastatic melanoma (strong staining): LIFr staining was predominantly in the cytoplasm; LIFr expression was significantly increased from nevi to melanoma. Bar = 10 µm. LIFr, leukemia inhibitory factor receptor; Nevi, normal nevi; PM, primary melanoma; MM, metastatic melanoma.

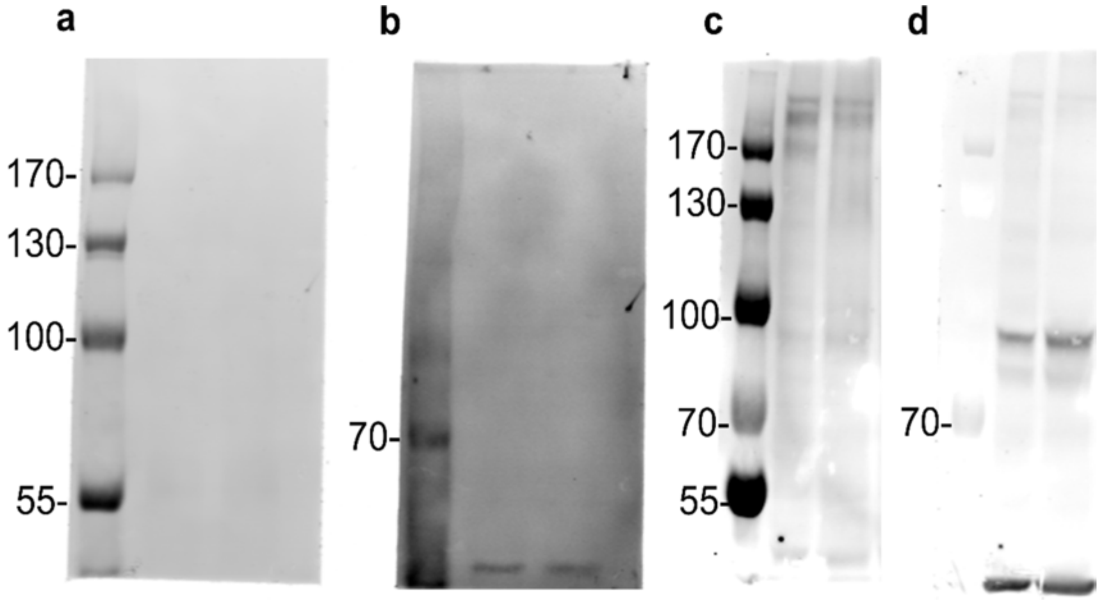

Supplementary Figure S2: LIFr antibody blocking test with Western blot to confirm the specificity of LIFr antibody. Whole-cell lysates were prepared from MMRU and PMWK cell lines. Cellular proteins were resolved on 8% SDS-PAGE gels and electrotransferred onto polyvinylidene difluoride membranes (Bio-Rad, Hercules, CA). Specific LIFr blocking peptides (LIFr C-19 P; Santa Cruz) were mixed at a 10:1 ratio with primary antibody and then incubated with the membrane overnight at 4°C. The bands around 190 KD disappeared after LIFr antibody neutralization **a.** but actin bands remained **b.** suggesting antibody specificity for the bands; LIFr expression was present as one or two bands around 190 KD **c.** while the actin band was present around 40 KD **d.** in melanoma cell lines.

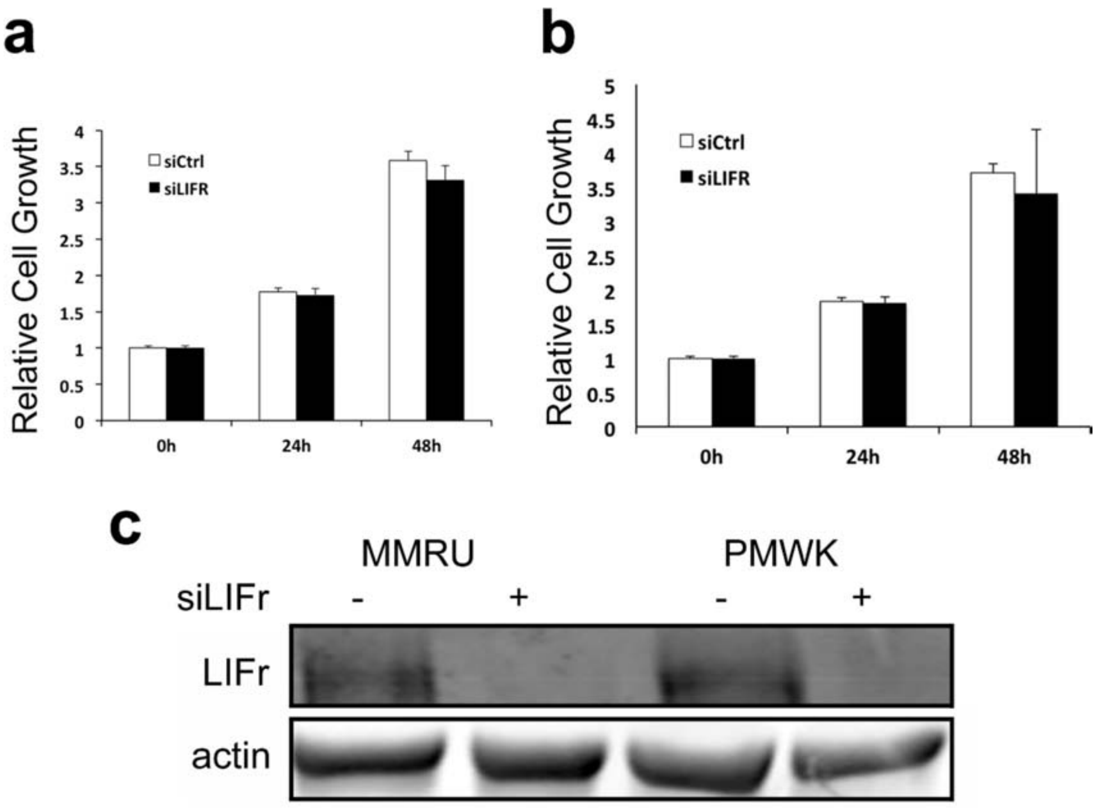

Supplementary Figure S3: Knockdown of LIFr has no significant influence on melanoma cell growth. MMRU cells **a.** and PMWK cells **b.** were transfected with LIFr siRNA and the cell growth was examined by SRB cell proliferation assay. The results are expressed as the mean value of triplicate samples, Bars equal to means ± SD. LIFr expression in MMRU cells from (a) and PMWK cells from (b) was examined by Western blot analysis **c.**

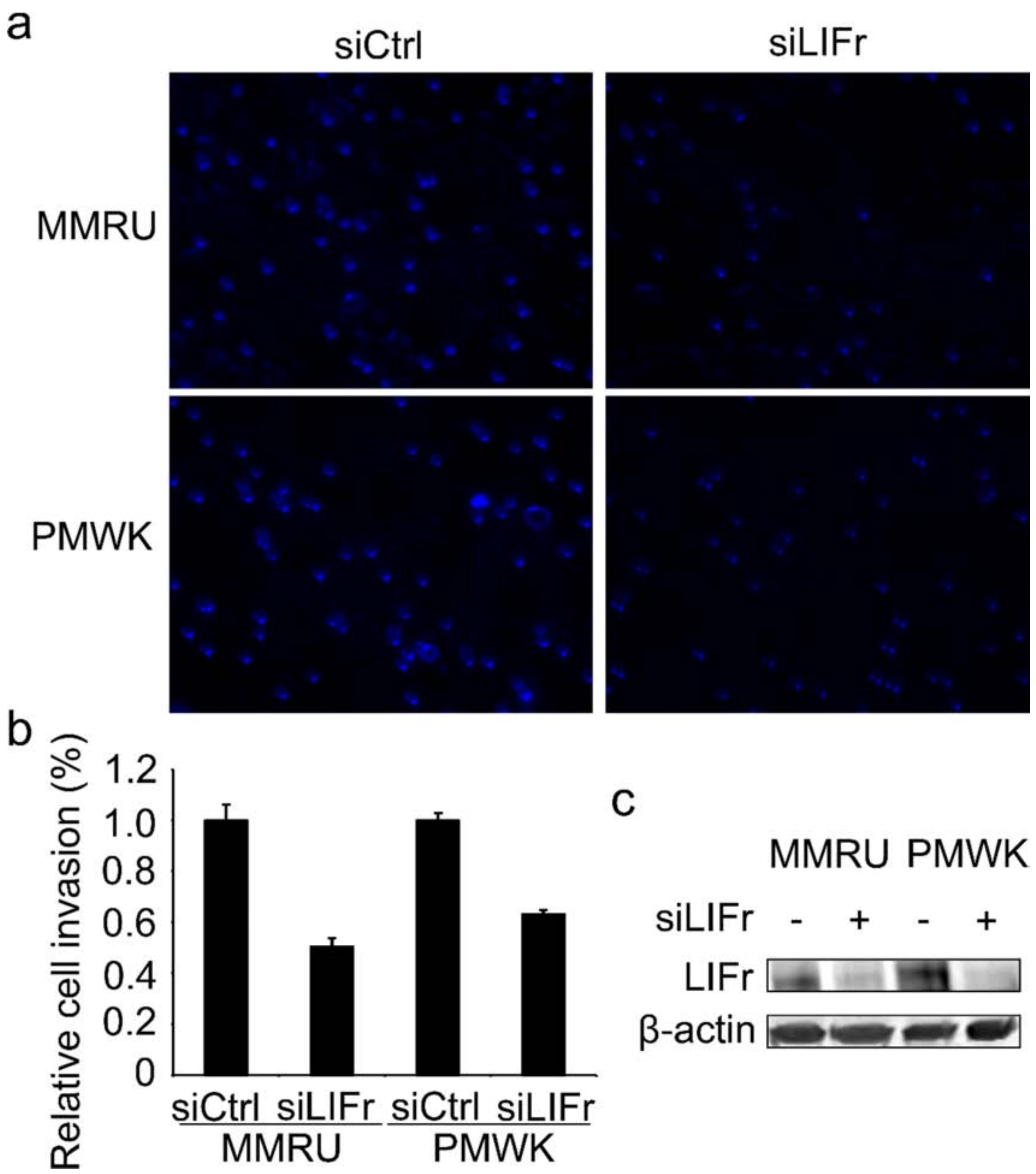

Supplementary Figure S4: LIFr knockdown tends to inhibit MMRU cell invasion. 48 h after MMRU and PMWK cell transfection, cell invasion assays were performed using Matrigel (BD Biosciences, Mississauga, Canada) applied to 24-well Transwell culture chambers. **a.** The invaded cells on the filter were counted under a Zeiss Axioplan fluorescence microscope (Carl Zeiss, Germany) or **b.** the retained dye on the filters was extracted by 30% acetic acid, followed by reading the absorbance at 590 nm. The dye-uptake assay was consistent with the cell counting assay. Bar represents the mean ± SD of three independent experiments. The experiments were performed in triplicates. **,$P < 0.01$. There was a trend that LIFr knockdown slowed down the MMRU cells' invasion, but did not reach statistical significance. **c.** Western blot analysis of LIFr expression in siLIFr RNA and siCtrl RNA transfected MMRU and PMWK cells.

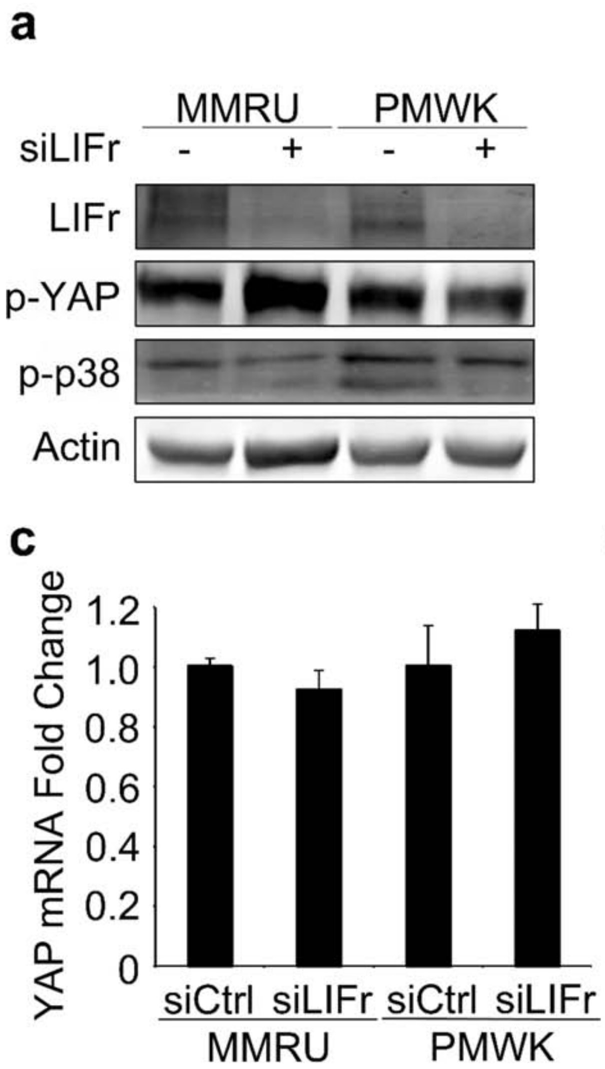

Supplementary Figure S5: Phosphorylated YAP was not modified by LIFr knockdown at both the mRNA and protein expression levels. Forty-eight hours after transfection, cells were harvested and examined for LIFr, p-YAP and p-MAPK(P38) mRNA and protein expression using qPCR and Western blot analysis. **a.** Western blot analysis of expression for LIFr, YAP, and MAPK; **b.** mRNA fold change of LIFr relative to the levels of control (siCtrl) cells (fold change = 1). **c.** qPCR analysis for YAP and MAPK (P38). The data was analyzed by Student's *t*-test. Bar equals to mean ± SD. All experiments were carried out in triplicate.
